# Comparative Analysis of CD4+ and CD8+ T Cells in Tumor Tissues, Lymph Nodes and the Peripheral Blood from Patients with Breast Cancer

**DOI:** 10.6091/ibj.1289.2014

**Published:** 2015-01

**Authors:** Farhad Riazi Rad, Soheila Ajdary, Ramesh Omranipour, Mohammad Hossein Alimohammadian, Zahir M. Hassan

**Affiliations:** 1 *Dept. of Immunology, Faculty of Medicine sciences, Tarbiat Modares University, Chamran Highway, Tehran, Iran; *; 2 *Immunology Dept., Pasteur Institute of Iran, Pasteur Ave., Tehran, Iran; *; 3 *Surgical Oncology Ward, Cancer Institute of Tehran University Of Medical Sciences, Gharib St., Tehran, Iran*

**Keywords:** Breast neoplasms, CD4+ T lymphocyte, CD8+ T lymphocytes, CXCL10, Granzymes

## Abstract

**Background:** CD4+ and CD8+ T cells are the main types of lymphocytes in cell-mediated immunity and play a central role in the induction of efficient immune responses against tumors. The frequencies of T cell subtypes in the peripheral blood and tumor tissues, and draining lymph nodes (dLN) can be considered as useful markers for evaluation of the immune system in cancers. **Methods:** In this study, the frequencies of CD4+ and CD8+ T cells in blood, tumor tissues, and dLN samples of breast cancer patients were compared with each other and with similar tissues from normal individuals. Immunophenotyping was carried out by flow cytometry and the expression levels of CXCL10, granzyme B, and mammaglobin were evaluated by real-time PCR. **Results: **In the peripheral blood, there were no differences in the T cell subsets between the patients and the normal individuals. The frequency of CD8+ T cells was significantly higher in tumor tissue than normal breast tissues while granzyme B expression was similar. Based on mammaglobin expression levels, dLN have been classified into micro- and macro-metastatic dLN. We found significantly lower frequency of CD4+ in macro-metastatic dLN than micro-metastatic dLN. CD8+ frequency was similar in both dLN; however, granzyme B expression was higher in micro-metastatic ones. There was not any significant difference in CXCL10 expression between the two types of dLN. **Conclusion:** Based on our results, although the tumor does not affect the systemic immunity, tumoral cells affect the local immune system in the tumoral tissues and the metastatic dLN.

## INTRODUCTION

Breast cancer is the most frequent type of cancer in women and an important public health problem worldwide [1, 2]. In Iranian women, breast cancer prevalence and mortality have been reported as 17.7 and 5.2 in 100,000, respectively [3]. The mean age range of breast cancer incidence is from 47.1 to 48.8 years in Iranian women that is at least one decade lower than the mean for women in the developed countries [4]. Several studies have documented that immune responses play an essential role in immunosurveillance and control of cancer cells [-]. These immune responses are predominantly mediated by the cell-mediated immunity. CD4+ and CD8+ T cells are the main types of lymphocytes in cell-mediated immunity and play a central role in the induction of efficient immune responses against tumors [8]. CD8+ T cells can recognize tumor antigens bound to class I MHC molecules on the tumor cells and directly kill them. On the other hand, the generation of tumor-specific cytotoxic T lymphocyte (CTL) responses is believed to depend on the help of activated CD4+ T cells, which recognize tumoral antigens presented with class II MHC molecules on antigen presenting cells. Some experiments have shown that in the absence of CD8+ T cells, CD4+ T cells can eliminate the tumor cells [9, 10]; however, effective tumor elimination needs both CD4+ and CD8+ T cells [11].

The establishment of adaptive immune responses to neoplasm involves at least three compartments namely, the peripheral blood, the draining lymph nodes (dLN), and the tumor tissues. There is evidence for changes in the frequencies of CD4+ and CD8+T cells in the peripheral blood and of the tumor tissues of breast cancer patients with controversial findings [12]. Moreover, the role and prognostic significance of tumor-infiltrating lymphocytes (TIL) within a tumor microenvironment is still debated.

Little is known about the phenotypes of T cells in the dLN of tumor tissues. Lymph nodes are the major components of the lymphatic system and are crucial sites for the initiation of the immune responses [13]. Meanwhile, they are also the preferential site of initial tumor metastases [14]. For patients with cancer, the dLN are commonly considered as prognostic indicators of the clinical outcome [15], and their immunological nature is often ignored. In the present study, to clarify the possible compositional significance of CD4+ and CD8+ T cells in the tumor tissues, the dLN and the peripheral blood, we determined CD4+ and CD8+ T cell percentages and CD4+/CD8+ ratios in these compartments and compared them with corresponding samples from healthy volunteers. Moreover, CXCL10, as a potent T cell-attracting chemokine, and granzyme B, as a marker of activated CD8+ T cells, were investigated. 

## MATERIALS AND METHODS

***Patients and healthy volunteers. ***Blood, tumor tissues, and dLN samples were collected from patients with breast cancer (n = 20; aged from 29 to 74 years), admitted to Emam Khomeini Hospital in Tehran, Iran. The study was approved by the Institutional Review Board of the Hospital, and an informed consent was obtained from all patients. Histologically, all patients had tumors classified as ductal carcinoma grades II and III. The range of the tumor size was from 2 to 5 cm. None of the patients had received chemotherapy, and all of them were negative for *human immune-deficiency virus*, *hepatitis C virus*, and *hepatitis B virus*. Normal healthy blood donors (n = 10) were recruited among the female laboratory coworkers (aged from 42 to 59), and normal breast tissues were obtained from healthy individuals (n = 10) who had mastoplasty for cosmetic purposes. We did not have access to normal lymph nodes. The tumoral and normal breast tissues and dLN samples were divided into two parts to perform flow cytometry and real-time PCR assays.

***Preparation of single cell suspension from breast tissues and dLN. ***Single cell suspensions from normal and tumoral breast tissues were prepared by enzymatic digestion as described by Whiteside and colleagues [16]. Briefly, after fat removal, blood or necrotic areas from the tumor samples as well as normal and tumor tissues were washed in RPMI 1640 (Sigma, MO, USA) the tissues were then cut into ~1 mm^3 ^pieces and digested with RPMI 1640, containing 100 U/ml penicillin, 100 µg/ml streptomycin, 200 IU/ml collagenase type IV (Sigma, MO, USA), and 0.02% DNase I (Sigma MO, USA) at 37^o^C for 30 min. The digested material was then passed through a mesh to remove clumps and the filtrate was washed twice, first time in the medium and the second time in PBS by centrifugation in room temperature at 400 ×g for 8 min. For preparation of single cell suspensions, after removal of fat and cutting the tissues into small pieces, the dLN were minced and passed through a mash to remove clumps and the filtrate was washed as above. 

***Flow cytometry analyses. ***Two-color flow cytometry was performed to determine CD4+ and CD8+ T cells. All antibodies were purchased from eBioscience, San Diego, CA, USA. Whole blood (100 µl with EDTA as anti-coagulant) was incubated with anti-CD4-PerCP-Cy5.5 and anti-CD8-phycoerythrin antibodies in the dark at 4^o ^C for 20 min. The RBC were then lysed by FACS Lysing Solution (BD Bioscience, CA, USA) and washed with PBS by centrifugation at 400 ×g for 8 min. For staining CD4+ and CD8+ T cells in the dLN and the breast tissues, single suspensions (5 × 10^5^) of these cells (as described above) were used for surface staining similar to the whole blood cells above, without using RBCs lysing. Flow cytometry was performed with Partec PAS III (Partec GmbH, Germany) flow cytometer, and the data were analyzed with FlowJo 7.6 (Tree Star Inc., CA, USA) software. Isotype-matched control antibodies were used to detect non-specific binding to the cells. 

***RNA extraction and real-time PCR. ***The tissues and the dLN samples were grinded to fine powder in the presence of liquid nitrogen to facilitate RNA extraction. Total RNA was extracted using QIAzol Lysis reagent (Qiagen, Germany) according to the manufacturer’s instructions. The quality and quantity of RNA were monitored using a NanoDrop1000 spectrophotometer (Thermo scientific, DE, USA). RNA was reversely transcribed using M-MULV (Fermentas, Germany) at 42^o^C for 1 h and also by a random hexamer (Fermentas, Germany) as primer. The amplifications were performed using a Rotor Gene 6000 (Corbett Research, Australia) thermocycler and Real Q-PCR 2× Master Mix Kit (Amplicon, Denmark) in 40 cycles. Each reaction contained 5-µl master mix, 100 nm primers for hypoxanthine phosphoribo-syltransferas, CXCL10, granzyme B, and mamma-globin, and 1 µl template cDNA (the primer sequences are shown in Table 1). Hypoxanthine phosphoribo-syltransferas was used as a reference gene for normal-ization of the gene expression. The expression level of mammaglobin (a mammary-specific protein) mRNA in the dLN was determined as a marker of metastasis. The efficiencies for primers used in the study varied between 95% and 105%. Moreover, melting curve analysis and agarose gel electrophoresis were performed to confirm the specificity of the primers. *Delta CT(Δ*Ct) was calculated using the following formula: [ΔCT = CT (target) – CT]. Gene expression level was determined by 2^-ΔCt^ method [17]. 

**Table 1:** primer sequences

**Gene**	**Sequences**	**Size** **(bp)**
HPRT	AATTATGGACAGGACTGAACGTCT TGCT	117
	TCCAGCAGGTCAGCAAAGAATTTATAGC	
		
CXCL10	GTCCACGTGTTGAGATDCATTGCTAC	163
	TGGAAGCACTGCATCGATTTTGCTC	
		
Granzyme B	AGACGACTTCGTGCTGACAGCTG	181
	CTCCAGCTGCAGTAGCATGATGYC	
		
Mammaglobin	CGG ATG AAA CTC TGA GCA ATG T	107
	CTG CAG TTC TGT GAG CCA AAG	

***Statistical analyses*** ***. ***Data were analyzed using by GraphPad Prism version 6.01 for Windows (GraphPad Software Inc, CA, USA). The significance of the results was calculated by student’s *t*-tests. *P* values of < 0.05 were considered significant. Data were expressed as mean and standard error of the mean (SEM).

## RESULTS

***Frequency and the ratio of CD4+ and CD8+ cells in the peripheral blood, the tumor tissues, and the dLN.*** The main types of lymphocytes in cell mediated immunity are CD4+ and CD8+ T cells, which play a central role in the induction of efficient immune responses against the tumor. Therefore, we compared the frequency of CD4+ and CD8+ T cells and the CD4+/CD8+ ratio between the peripheral blood and the breast tissues and also between the cancer patients and the healthy controls using flow cytometry. 

**Fig. 1:**
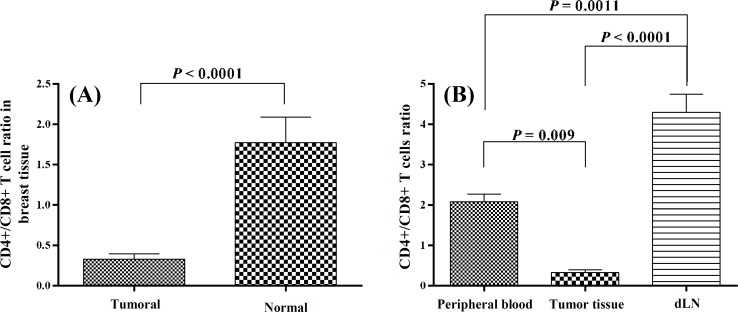
The ratios of CD4+/CD8+ cells. **(A)** The CD4+/CD8+ ratios compared between the tumor and normal breast tissues. **(B) **The CD4+/CD8+ ratios among the peripheral blood, the tumor tissue and the dLN. dLN, draining lymph nodes

In the peripheral blood, there was no difference in the mean frequencies of CD4+ and CD8+ T cells and the mean CD4+/CD8+ ratio between the cancer patients and the healthy controls (Table 2). However in the breast tissues, the mean frequency of CD8+ T cells was significantly higher in the tumor tissues (10.24 ± 1.9) compared to the normal breast tissues (3.75 ± 1.4; *P *= 0.034). Data analyses revealed that although the mean frequency of CD4+ T cells was lower in the tumor tissues compare to the normal tissues, the difference was not significant. The increase in CD8+ T cells caused a significant decrease in CD4+/CD8+ ratio of the tumor tissues compared to the control tissues (*P*<0.0001, Fig. 1A and Table 3). There were strong significant negative correlations between both CD4+ and CD8+ T cells and the size of the tumors (*P *= 0.07, r = -0.54 and *P *= 0.02, r = -0.66, respectively). The mean frequency of CD4+ and CD8+ T cells in the dLN were 44.1 + 3.4 and 11.1 + 1, respectively. 

The comparison of the CD4+/CD8+ ratio between the peripheral blood, the tumor tissues, and the dLN of the patients’ samples indicated that the ratio for the dLN (4.3 ± 0.45) was significantly higher than those for the peripheral blood (2.08 ± 0.19, *P* = 0.001) and the tumor tissues (0.33 ± 0.07, *P*<0.0001). This ratio was also significantly higher in the peripheral blood in comparison to the tumor tissues (*P*=0.009, Fig. 1B). Interestingly, the mean CD4+/CD8+ ratio in the peripheral blood (2.8 ± 0.5) and the breast tissues (1.8 ± 0.5) was comparable in normal volunteers.

**Table 2:** T cell subsets in the peripheral blood from breast cancer patients and the healthy controls

**Subpopulations**	**Patients (n = 20)**			**Healthy controls (n = 10)**			***t*** ** test**
	**Mean ± SEM**	**CI %95**		**Mean ± SEM**	**CI %95**		***P*** ** value**
%CD4+ in lymphocytes	35.6 ± 1.5	32.4-38.8		39.7 ± 1.8	35.6-43.8		ns
%CD8+ in lymphocytes	20.5 ± 2.3	15.7-25.2		18.4 ± 3.8	9.8-26.9		ns
CD4+/CD+ ratio	2.1 ± 0.2	1.7-2.5		2.8 ± 0.5	1.8-3.9		ns

SEM, standard error of the mean; CI, confidence interval

**Fig. 2:**
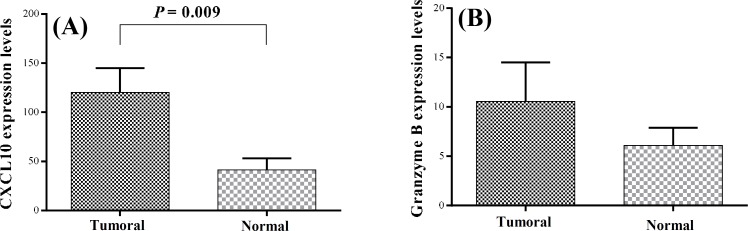
The expression of CXCL10 and granzyme B mRNA levels in breast tissues**. (A)** CXCL10 and **(B)** granzyme B mRNA expression levels compared between the tumoral and the normal breast tissues evaluated by real time PCR.

***Expression of CXCL10 and granzyme B in the tumoral and normal breast tissues and the dLN. ***Due to our obtained results that indicated the increased frequencies of CD8+ T cells in the tumor tissues and high CD4+/CD8+ ratio in the dLN, we decided to study the expression of CXCL10, as an important chemotactic factor for T cells and also granzyme B, as an effector molecule for CD8+ T cells function, in the tumoral and normal breast tissues and dLN of the patients. Real-time PCR analysis showed that the mean expression levels of CXCL10 (120.2 ± 24.7) in the tumor tissues were significantly higher than the control (41.4 ± 11.6; *P *= 0.009). However, granzyme B expression levels in the tumor tissues (10.6 ± 4) were not significantly higher than the controls (6.1 ± 1.8). The mean expression levels of CXCL10 and granzyme B in the dLN were 91.3 + 18.6 and 5.2 + 0.8, respectively. Moreover, there were no correlations among the frequencies of the T cell subsets, CXCL10, and granzyme B mRNA expressions neither in the tumor tissues nor in the dLN. Similarly, there were no significant differences in CXCL10 and granzyme B mRNA expressions between the tumor tissues and the dLN (Fig. 2).

**Fig. 3:**
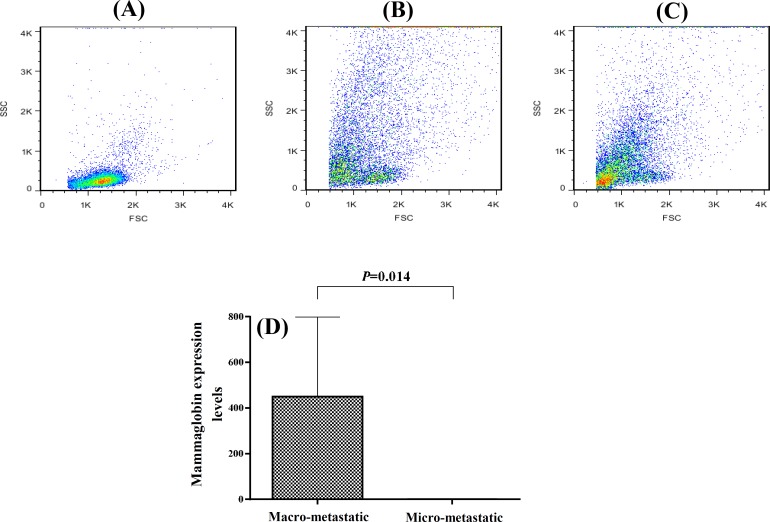
The cell distribution pattern and mammaglobin mRNA expression levels in macro- and micro-metastatic dLN. Representative flow cytometric patterns of the cells shown in micro-metastatic dLN **(A)**, in the macro-metastatic dLN **(B)**, in the tumor tissues **(C)**. Mammaglobin mRNA expressin levels compared in micro- and macro-metastatic dLN by real time PCR **(D)**. dLN, draining lymph nodes

**Fig. 4:**
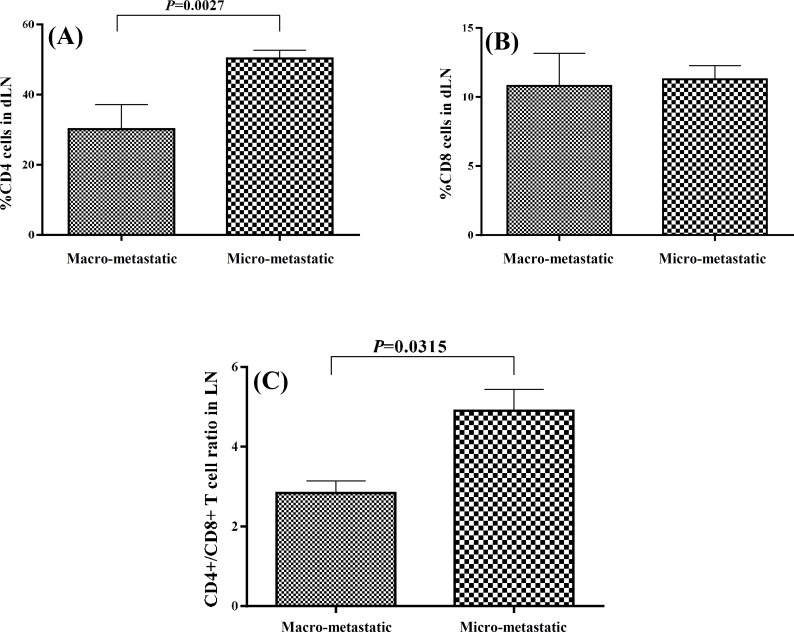
T cell subsets frequencies in dLN. The T cell subsets frequencies compared between the macro- and micro-metastatic dLN (**A** and **B)**. The CD4+/CD8+ ratio compared between the macro- and micro-metastatic dLN **(C)**. dLN, draining lymph nodes

**Fig. 5:**
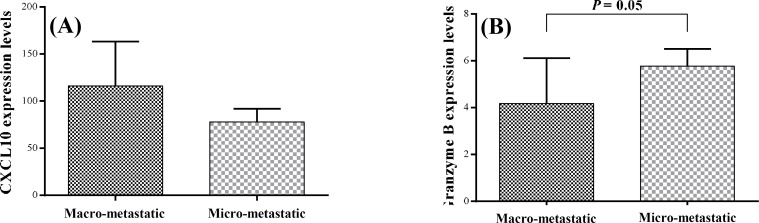
The expression of CXCL10 and granzyme B mRNA in the dLN.** (A)** CXCL10 mRNA expression levels and **(B)** granzyme B mRNA expression levels compared between the macro- and micro-metastatic dLN. dLN, draining lymph nodes

***Comparison of T cell subsets and gene expression in macro-metastatic and micro-metastatic dLN. ***The tumor dLN are the first sites that tumor metastasis occurs. For evaluation of the effects of tumor spread to the dLN on the T cell subsets and also the function of the immune system, it was first necessary to detect the metastasis in the dLN. Therefore, we analyzed the expression levels of mammaglobin mRNA in the dLN, as a reliable and accurate marker of metastasis [18], using real-time PCR. We considered mammaglobin expression levels of more than 1 as macro-metastatic (n = 6) and the expression levels of less than one as micro-metastatic (n = 11). The macro-metastatic dLN had very high mammaglobin expression levels (450.2 ± 348.2) while the mean ± SEM mammaglobin expression in micro-metastatic dLN was 0.17 ± 0.06. Moreover, as depicted in Figure 3, the tumor cells were the predominant cell population in the macro-metastatic dLN, whereas lymphocytes were the predominant cells in the micro-metastatic dLN. The frequency of CD4+ T cells and CD4+/CD8+ ratio were significantly lower in the macro-metastatic dLN (30.2 ± 7 and 2.8 ± 0.3, respectively) as compared with the micro-metastatic ones (50.4 ± 2.3 and 4.9 + 0.5, respectively; *P*≤0.03, Fig. 4). The mean frequency of CD8+ T cells was similar in both dLN groups. Although the mean expression levels of CXCL10 mRNA in macro-metastatic dLN (116.1 ± 47.1) were higher than the micro-metastatic ones (71.8 ± 14.2), the difference was not significant. However, the real-time PCR results indicated a significant difference in granzyme B expression between the macro-metastatic dLN (4.2 ± 1.9) and the micro-metastatic ones (5.8 ± 0.7; *P=*0.05, Fig. 5). 

**Table 3:** T cell subsets in tumoral and normal tissues

**Subpopulations**	**Tumoral tissue (n = 16)**			**Normal tissue (n = 10)**			***t*** ** test**
	**Mean ± SEM**	**CI %95**		**Mean ± SEM**	**CI %95**		***P*** ** value**
%CD4+ in lymphocytes	2.9 ± 0.6	1.7-4.2		7.4 ± 4.0	3.8-18.5		ns
%CD8+ in lymphocytes	10.2 ± 1.9	6.1-14.4		3.8 ± 1.4	0.2-7.7		0.034
CD4+/CD+ ratio	0.3 ± 0.1	0.2-0.5		1.8 ± 0.5	0.2-7.7		<0.0001

SEM, standard error of the mean; CI, confidence interval

## DISCUSSION

The immune system plays an important role in the pathogenesis and progression of the breast cancer. CD4+ and CD8+ T cells are the main types of lymphocytes in cell-mediated immunity and play a central role in the anti-tumor immune responses [19]. Several studies have reported the changes in the frequency of CD4+ and CD8+ T cells and the CD4+/CD8+ ratio in the peripheral blood of patients with different types of cancers. Reversed CD4+/CD8+ ratios due to reduced percentage of CD4+ T cells have been reported in patients with colon, gastric, and esophageal cancers; however, studies on other types of cancer have found normal CD4+/CD8+ ratios [-]. In this study, we did not find any significant differences in CD4+ and CD8+ T cells and CD4+/CD8+ ratio of the peripheral blood between the breast cancer patients and the controls. This finding is in line with the findings of Schroder *et al*. [23] who did not report any significant differences in CD4+ and CD8+ T cells and the CD4+/CD8+ ratio between breast cancer patients and controls in their study.

TIL in human cancers may play an important role in anti-cancer immunity. TIL have been suggested to be a manifestation of host immune reactions to cancer cells [24]. The shift in CD4+/CD8+ ratio of TIL in different cancers can be due to the declined CD4+ T cells and/or to the increased CD8+ T cells [21]. Reversed CD4+/CD8+ ratios have been correlated with different outcomes in various cancers. For instance, decreased proportions of tumor-infiltrating CD4+ T cells with reversed CD4+/CD8+ ratios were highly correlated with rapid tumor growth and lymph node metastasis in cervical carcinoma [25]. In contrast, high CD4+/CD8+ ratio due to high percentage of CD4+ cells in tumors have been correlated with lymph nodes metastasis and reduced patient survival in breast, renal, esophageal and small cell lung carcinomas [24, 26].

Most studies have evaluated the composition of TIL by *in situ* immunohistochemistry [24, 27, 28]. Here, we used flow cytometry, which is a more accurate technique. Our results indicated no significant difference in CD4+ T cells frequency in the tumor tissues compared to the normal ones compared to the normal tissues results, the mean percentage of CD8+ T cells in the tumor tissues was significantly higher. Therefore, the CD4+/CD8+ ratio was inverted in favor of CD8+ T cells. The low CD4+/CD8+ ratio is consistent with the results obtained by Laguens *et al*. [27] who reported lower ratios of CD4+/CD8+ in tumor tissues compared to normal tissues; nevertheless, they reported increases in both CD4+ and CD8+ cells in the breast tumor tissues compared to the normal tissues. We found strongly significant negative correlation between the frequency of both CD4+ and CD8+ T cells in the tumor tissues and the size of the tumors; i.e. the higher the T cells, the lower the tumor size. Moreover, this finding confirms the studies that have indicated an association between the frequencies of the T cells and the improvement in the clinical outcome [24, 29]. 

Our results indicated that the CD4+/CD8+ ratio was significantly different in the peripheral blood, the tumor tissues, and the dLN in patients with breast cancer. The CD4+/CD8+ ratio was the highest in the dLN, followed by the blood and the tumor tissues. It seems that the tumors can actively change the frequency of the T cell subsets in their micro-environments. 

CXCL10 is a potent chemokine for the recruitment of CD4+ and CD8+ T cells into the tumor sites [30]. Increased CXCL10 mRNA expression has been reported in hepatocellular carcinoma and was correlated with the number of infiltrated lymphocytes [31]. Considering the changes in the frequency of T cell subsets in patients with tumor, we studied the expression of CXCL10 mRNA and found significant increases in the expression of this chemokine in the tumor sites. However, CXCL10 expression did not correlate with the frequencies of CD4+ and CD8+ T cells. Thus, it seems that other factors may also influence the T cell recruitment to the tumor site. 

The tumor dLN are the first sites where antigens are presented to naive lymphocytes in order to elicit anti-tumor immune responses. Meanwhile, these dLN are the preferential sites for initial tumor metastasis. Investigating the immunological status of the dLN is a very important issue in tumor immunology. However, dLN are frequently used to predict patient outcome, and survival and their immunological nature have not been well investigated [15]. The local micro-environments in the dLN can affect the subsequent immune responses toward the tumor [14]; therefore, their accurate identification as metastatic or non-metastatic is deemed essential. This identification is often carried out by conventional histological methods. However, the small number of the tumor cells in dLN often leads to false-negative histology results. Mammaglobin expression is a sensitive molecular marker for the detection of micrometastasis in the tumor dLN. Real-time PCR assays targeting mammaglobin mRNA, have been introduced as sensitive and specific methods to identify the presence of tumor cells in dLN [32]. It has been shown that mammaglobin mRNA is detectable in metastatic lymph nodes from patients with breast cancer, whereas its expression in uninvolved lymph nodes is not detectable [33]. 

We analyzed the expression levels of mammaglobin mRNA in the dLN by real-time PCR. All lymph nodes, even those identified histology as non-metastatic, were positive for mammaglobin mRNA expression. Based on mammaglobin mRNA expression levels, we divided the dLN as micro-metastatic (mRNA expression levels less than one) and macro-metastatic (mRNA expression levels more than one). To determine whether the micro-metastatic and the macro-metastatic dLN from patients with breast cancer are immunologically different, the frequency of CD4+ and CD8+ T cells and CD4+/CD8+ ratios was determined. The macro-metastatic dLN exhibited significantly lower CD4+ T cells and CD4+/CD8+ ratio compared with the micro-metastatic ones. The mean frequency of CD8+ T cells was similar in the both dLN groups. Therefore, it seems that the presence of a high number of tumor cells in the lymph node may diminish CD4+ cells. Evaluation of CXCL10 mRNA expression in the micro- and macro-metastatic dLN revealed similar expression in the both dLN groups. Consistent with our results, it has been shown that the CD4+/CD8+ ratio in dLN was decreased with metastasis of tumor cells in breast cancer patients [34]. In another study, CD4+/CD8+ ratio has been studied in non-metastatic dLN and an inverted ratio has been reported [35]. In these studies, the metastatic statuses of the lymph nodes have been determined by histological methods. It is noteworthy that the decreased CD4+/CD8+ ratio in the tumor tissues is accompanied by increased frequency of CD8+ T cells while in the dLN, this decline is coincided with decreased frequency of CD4+ cells. The significance of this finding remains to be elucidated. 

To assess the effector function of CD8+ T cells, we studied the expression of granzyme B in the tumoral and the normal breast tissues and the dLN. Granzymes are known to be highly expressed in terminally differentiated CD8+ T cells [36]. So far, 11 members of the granzyme family have been found in humans, but granzyme B is the only member of this family that has a proteolytic cleavage after aspartate residue to induce apoptosis. Hence, granzyme B is the most important granzyme in the CD8+ T cells function [36]. Although the mean mRNA expression of granzyme B in the tumor tissues was higher than that of normal breast tissue, the difference was not significant. This is consistent with a study that has shown the number of granzyme B positive cells is reduced in TIL of breast and lung cancer patients [37]. In viral infections, chronic stimulation leads to the loss of effector CD8+ T cells function by down-regulation of perforin and granzyme B expressions [38]. Similar chronic stimulation of the immune cells in the tumor microenvironments may cause alterations in CD8+ T cell function [39]. Thus, despite the increased frequency of CD8+ T cells in the tumor site, it seems that there is a defect in the cytotoxic function of these cells. The real time-PCR results indicated a significantly higher granzyme B expression in micro-metastatic dLN compared to the macro-metastatic ones. This finding suggests the presence of more functional CD8+ cells in micro-metastatic dLN and may explain the low tumor cell presence in these lymph nodes. 

In conclusion, our results revealed that the CD4+/CD8+ ratios in the peripheral blood and breast tissues from the normal controls were similar. This ratio was lower in the tumor breast samples due to the high CD8+ T cell percentage in these tissues. Despite this, similar frequency of granzyme B mRNA expression levels in the tumor and the normal tissues indicated that CD8+ T cells in the tumors were not active. Moreover, based on mammaglobin mRNA expression levels, we divided dLN into micro- and macro-metastatic groups and found lower CD4+/CD8+ ratios in the macro-metastatic ones. Interestingly however, the expression of granzyme B mRNA was higher in the micro-metastatic dLN, indicating the presence of a more functional CD8+ T cell population in micro-metastatic dLN which may control the metastatic tumor cells in these compartments. Collectively, these data point that breast cancer is associated with dysregulation of T cell subsets in the dLN and the tumor tissues, which together may play crucial roles in the immunopathogenesis of this malady.
